# The effect of an interactive game-based e-book with simulative clinical scenarios on the health literacy competency among medical students in Taiwan

**DOI:** 10.1186/s12909-025-07541-9

**Published:** 2025-07-01

**Authors:** Pei-Ling Tseng, Hui-Fang Yang, Chia-Chen Chang, Tai-Yuan Chiu, Jaw-Shiun Tsai, Hsiang-Ru Lai, Chen-Yin Tung

**Affiliations:** 1 Department of Health Promotion and Health Education, College of Education, National Taiwan Normal University, Taipei, Taiwan; 2 Department of Family and Community Medicine, Tri-Service General Hospital, National Defense Medical Center, Taipei, Taiwan; 3 Ministry of Health and Welfare, Taipei, Taiwan; 4 Department of Family Medicine, National Taiwan University Hospital, Taipei, Taiwan

**Keywords:** Health literacy, Medical students, Clinical scenarios, Interactive E-book, Simulation

## Abstract

**Background:**

Health literacy plays a vital role in effective patient-physician communication. Over the past decades, health literacy efforts have strengthened public empowerment by improving access to health information and its effective use. Despite this progress, there are still insufficient digitalized teaching materials in medical schools, especially in the post-pandemic era. To address this gap, this study developed an interactive e-book as a supplementary e-learning tool for fifth-year medical students during their family medicine clerkship. The e-book integrates game-based learning, multimedia content, and scenario-based simulations to enhance students’ knowledge, attitudes, and skills in health literacy.

**Methods:**

A true experimental design was employed, involving 216 medical students randomly assigned to experimental (*n* = 110) and control (*n* = 106) groups. Students in the experimental group utilized the e-book alongside standard clerkship training, while the control group participated in standard training alone. Pre- and post-intervention assessments measured health literacy knowledge, attitudes, and skills using a validated questionnaire.

**Results:**

Results showed the experiment group (*n* = 110) who read the e-book demonstrated greater improvements in health literacy knowledge, attitude, and skills than the control group (*n* = 106). Knowledge scores increased by 0.117 points, attitudes by 0.175 points, and skills by 0.162 points (*p* < 0.05). Students reported high satisfaction with the e-book’s engaging and interactive format, highlighting its potential for enhancing learning outcomes.

**Conclusions:**

This study demonstrates the effectiveness of incorporating interactive digital tools into medical education to foster health literacy competencies, which should be expected in medical education and clinical pratical settings.

**Supplementary Information:**

The online version contains supplementary material available at 10.1186/s12909-025-07541-9.

## Introduction

Health literacy refers to individuals’ ability to make informed judgments and decisions regarding healthcare, disease prevention, and health promotion in their daily lives to maintain or enhance their quality of life [1]. Due to its impact on individuals’ comprehension of health information and decision-making in health-related matters, health literacy is considered a critical factor in medical communication, health education, and the prevention of medical disputes within the medical field [2]. Physicians, as the primary providers of medical resources, benefit from high health literacy by understanding and distinguishing between medical literacy and health literacy. This enables them to apply appropriate communication techniques to support individuals’ self-management of their health [3]. A survey conducted by Coleman from 2012 to 2016 on health literacy curricula in American medical schools revealed that over 80% of programs offering related courses focused solely on the concept of health literacy and the conditions faced by individuals with low health literacy. While physicians may consider themselves as highly health-literate, many skills acquired during training were difficult to apply in clinical practice [4–6].

To address this, physicians must develop health literacy competence through targeted training during medical education. Health literacy competence is defined as the ability to deliver medical information, treatments, and recommendations tailored to a patient’s health literacy level. This competence encompasses knowledge, attitudes, and skills. However, related courses in medical education in Taiwan are currently insufficient. Equipping future physicians with the right knowledge, skills and attitudes will enable more effective communication with patients during time-limited consultations, fostering patient well-being and positive patient-physician relationships.

In the technology-driven 21st century, digital learning (e-learning) has emerged as a global trend, with its significance further heightened by the impact of the COVID-19 pandemic. Consequently, countries worldwide are actively advancing digital learning through research and innovation. E-learning in medical education includes a wide range of instructional formats such as asynchronous modules [7], virtual patient simulations [8], flipped classrooms [9, 10], and mobile applications [11]. These methods provide flexibility, accessibility, and opportunities for active learning that traditional lectures lack. Recent studies emphasize the value of incorporating multimedia, interactive exercises, and self-paced feedback mechanisms in digital learning environments, which have been shown to enhance student engagement, motivation, and long-term knowledge retention. Studies have shown that digital learning tools featuring multimedia, self-paced content, and interactive feedback mechanisms can enhance students’ motivation, concentration, and learning outcomes [12–14]. Among digital learning methodologies, game-based learning has shown particular promise, imposing a lower cognitive load than non-game-based learning [15]. Numerous studies have reported that game-based digital learning can enhance motivation in medical education, improve attention and focus, enhance self-efficacy and health-related behaviors, provide individualized feedback, and offer personalized learning progress with opportunities for repetition [16, 17].

Interactive e-books are especially promising because they integrate textual, visual, and auditory content with methods like game-based learning—which improves attention, self-efficacy, and motivation—and scenario-based learning, which simulates clinical decision-making to enhance practical skills. While there are currently no health literacy courses in Taiwan that adopt this type of interactive e-book format, this gap is not unique to Taiwan. A recent systematic review has indicated that, over the past decade, health literacy education in higher education has been limited globally, with most curricular efforts primarily found in U.S. medical and pharmacy programs [18]. Moreover, e-book-based teaching in medical education is primarily used for clinical skills [19–21] or exam preparation [22], with few initiatives addressing health literacy communication training through digital simulation-based formats. Therefore, this study developed an innovative interactive e-book incorporating scenario-based and game-based learning approaches, specifically tailored to enhance health literacy competencies among medical students. The interactive e-book features the 4 A learning characteristics (Anytime, Anywhere, Anyone, Anyway) [23] and contains 12 clinical scenarios vividly enacted by real actors, portraying authentic patient-physician interactions. Interactive elements within the e-book include matching games, multiple-choice questions, masking stickers, supplementary information, and embedded Google Forms to track student learning progress in real-time. In addition, the e-book incorporates scenario-based simulation videos that portray realistic patient-physician interactions, allowing learners to observe, analyze, and reflect on appropriate communication techniques in clinical contexts.

This interactive e-book was accessed via the **eBookHub**^™^ app and utilized as an experimental intervention during clinical rotations for fifth-year medical students in the Family Medicine Department. The primary aim of this study was to evaluate the effectiveness of this innovative instructional module in improving the health literacy compentence of medical students. Specifically, the goals were to enhance students’ ability to rapidly assess patients’ health literacy levels, facilitate effective patient-physician communication characterized by acceptance and respect, and actively involve patients in medical decision-making processes.

## Method

### Participants

Patient-physician interactions are a critical component of family medicine residency training, with attending physicians often serving as key instructors in medical humanities courses. For this reason, this study specifically focused on the family medicine specialty. The study obtained consent from the family medicine departments of two medical universities in Northern Taiwan, both affiliated with teaching hospitals, to incorporate the designed educational material into their fifth-year family medicine clerkship curriculum. Each student participated in the program for a four-week period. The study was conducted from August 2021 to May 2022, during which campuses in Taiwan remained open for regular in-person classes [24].

The family medicine clerkship program was conducted in subgroups, each consisting of 8 to 15 students, and was implemented as a monthly rotation in the teaching hospitals. Only students who voluntarily provided informed consent after receiving study information participated in the questionnaire and were included as research subjects.

**Fig. 1:**
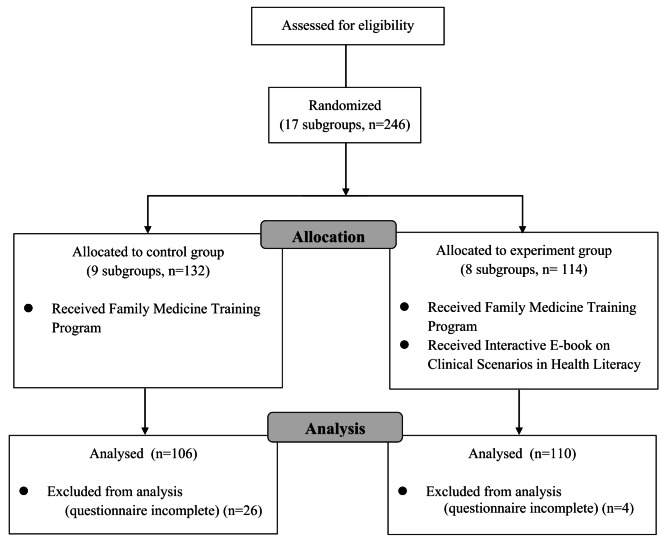
Flowchart of students’ enrollment and assessment

A total of 246 students were initially recruited for this study. Only students who completed the intervention program and both the pre- and post-intervention questionnaires were included in the analysis, resulting in 216 valid responses and an effective response rate of 87.8%. To prevent students in the experimental group from sharing course content with those in the control group, baseline data were first collected from the control group. This approach aimed to minimize information contamination between the experimental and control groups, thereby preserving the internal validity of the study findings. Data from the control group (*N* = 132) were collected in 2021, while data from the experimental group (*N* = 114) were collected in 2022. A detailed flowchart illustrating the study process is presented in Fig. 1.

### Experimental context and e-book development

During the family medicine clerkship at the two collaborating hospitals in this study, medical students were exposed to training program courses that covered various topics, including physical assessment, holistic care, occupational medicine, comprehensive geriatric assessment, patient-physician communication, case discussions, medical record writing, health behavior education techniques, chronic disease education, and discharge planning services. The family medicine training program was completed after an intensive four-week course, with minimal emphasis on health literacy. Therefore, this study developed an instructional e-book focused on health literacy for patient-physician interactions as an supplementary teaching resource, aiming to enhance medical students’ health literacy, promote positive patient-physician relationships, and improve healthcare quality.

Based on the health literacy module developed by Yang et al. (2022) [25], this study combined the Cognitive Theory of Multimedia Learning [26], Concept Mapping [27], and the Motivation Model [28] to design an interactive e-book titled *Interactive E-book on Clinical Scenarios in Health Literacy*. This innovative resource includes a comprehensive lecture covering the definition and practical applications of health literacy, interactive games, clinical consultation scenario videos, and related questions. With an approximate viewing time of 50 min, the e-book is structured to enhance learning outcomes through a systematic approach. The e-book’s objectives align with the core indicators of a physician’s professional competence in health literacy: (1) Concepts and assessment, (2) Acceptance and respect, (3) Communication and interaction, and (4) Medical information and decision-making [29].

The content of the e-book is delivered across two key sessions. The first session, addressing the first two indicators, introduces the foundational concepts of health literacy, its significance, and methods for evaluation. It incorporates interactive online tools such as multiple-choice questions, matching activities, and augmented learning tools to enable students to monitor their learning progress in real-time. Additionally, this session features a set of clinical consultation scenario videos, which simulate realistic patient-physician interactions. These videos are enacted by licensed physicians and trained actors portraying patients and are structured into five phases: (1) assessing the patient’s health literacy level, (2) identifying patient needs with acceptance and respect, (3) communication and interaction, (4) confirming patient understanding, and (5) medical information exchange and shared decision-making. For each phase, students are presented with two contrasting physician response scenarios and are prompted to select the most appropriate and effective communication strategy, thereby actively engaging in interactive simulation-based learning.

The second session, aligned with the third and fourth indicators, emphasizes new guidelines for effective patient-physician communication, covering verbal and non-verbal interactions, the use of simplified educational materials, and the shared decision-making process. To reinforce these concepts, the course provides 12 engaging simulation videos. After watching each video, students complete interactive card-matching activities that simulate real-life consultation decision-making, enabling them to apply and reinforce the communication strategies presented.

The game-based learning elements embedded in the e-book include interactive games such as matching exercises, multiple-choice quizzes with immediate feedback, masking-sticker challenges (e.g., “dot-to-reveal” or “scratch-and-learn” formats), and interactive card-matching scenarios that simulate real-life consultation decision-making. These elements are purposefully designed to sustain learner engagement, encourage repeated practice, and strengthen memory retention through active participation. Multimedia features include animated narrations, voiceovers, interactive scenario videos with authentic dialogue, and pop-up windows that provide contextual feedback or supplemental explanations. These multimedia features are integrated to create immersive, lifelike clinical encounters that reinforce communication techniques and decision-making skills. For detailed descriptions of the theoretical foundation and instructional features of the e-book, please refer to Table 1.

**Table 1:** The features of “interactive e-book on clinical scenarios in health literacy”

Item	Feature and Content Diagram	Corresponding Theory
1	A. Provide learners with multiple self-learning functions. B. Design concise texts, interesting images, vivid audio, and animated narration. C. Present corresponding texts and illustrations on the page or screen. D. Enhance learning effectiveness by incorporating interactive question games.	*Cognitive Theory of Multimedia Learning*: • Organize information • Principle of consistency • Principle of form • Principle of proximity • Principle of repetition
2	A. Through contextual simulation, medical students are able to acquire prerequisite knowledge in health literacy. B. Provide multiple derived new knowledge. C. Simulated demonstration-based learning.	*Concept Mapping*: • The simulative performance of health literacy in various healthcare contexts is visually expressed through graphic images to convey its conceptual content, accompanied by explanatory analysis within this concept.
3	A. Attractive color, font, and tone. B. Design book introductions and learning objectives to provide comprehensiveness and relevance. C. Set up vivid and interesting simulative clinical scenarios to stimulate learners’ interest.	*The Motivation Model*: • Focus • Relevance • Self-confidence • Satisfaction

The interactive e-book was developed over a rigorous six-month process, guided by instructional design principles [29]. The e-book underwent comprehensive pilot testing involving iterative evaluations by five experts and two fifth-year medical students, who assessed content coherence, interactive functionality, and visual clarity. This process led to refinements such as improved navigation, adjustable multimedia playback speeds, and enhanced interactive elements. Content validity was rigorously assessed, achieving a high overall Content Validity Index (CVI) of 0.93. Additionally, 41 fifth-year medical students participated in a pilot test, and the results demonstrated statistically significant improvements in students’ health literacy following the educational intervention. Specifically, knowledge scores increased from a pre-test correct rate of 63.0% to a post-test rate of 76.9% (*p* = 0.001); attitude scores improved from 4.51 to 4.82 out of 5 (*p* = 0.000); and skills scores increased from 4.11 to 4.66 out of 5 (*p* = 0.002), as measured by paired t-tests.

### Design and procedure

This study employed a true experimental design. Medical students were randomly assigned to several subgroups before they started their family medicine clerkship. Although the subgroup allocation was predetermined by administrative staff at the participating institutions, the assignment was independent of the students’ academic performance, personal preferences, or any baseline characteristics. Students were automatically assigned to one of 17 subgroups for their family medicine clerkship before the intervention. These subgroups were then allocated into experimental and control groups by the researchers according to the semester in which the clerkship occurred. This arrangement allowed for temporal separation between conditions and minimized potential cross-contamination. While this method did not involve individual-level randomization, the allocation of intact subgroups based on pre-existing schedules constitutes a cluster-level random assignment. Therefore, this study qualifies as a true experimental design with subgroup-level randomization. In addition to receiving training courses, the students in the experimental group also participated in the intervention using this e-book. The control group did not receive additional learning activities during the intervention period. The intervention process included the following steps: (1) In the first week of the clerkship, the physician-teacher explained the research objectives to the students and conducted an online questionnaire session on physicians’ health literacy professional competence (pre-test). The students were then instructed to view the e-book independently. (2) At the end of the clerkship, students had to complete a satisfaction survey within the e-book to confirm they had finish the reading. After ensuring all students had completed the survey, a classroom feedback and demonstration activity was conducted. (3) After evaluating students’ performance, students completed a post-test administration of the “physicians’ health literacy professional competence questionnaire” to evaluate changes in knowledge, attitudes, and skills. The control group students only participated in the family medicine clerkship training program. They completed the same pre-test in the first week and the post-test in the final week of the clerkship using the same questionnaire.

### Questionnaires

The “physicians’ health literacy professional competence questionnaire” used in this study was adopted from the evaluation questionnaire developed by Yang et al. (2022) [25]. It includes medical simulations that depict patient-physician interactions and is utilized as both a pre-test and post-test evaluation to assess the effectiveness of this instructional intervention. The questionnaire was divided into knowledge, attitude, and practice. The first set of questions evaluated the knowledge of leveling patients’ health literacy. The second set of questions measured the patience and willingness of doctors to communicate with patients at different levels of health literacy. The last set of questions assesses how well doctors can respond to patients with different levels of health literacy and make appropriate medical decisions.

This online questionnaire consists of 47 items, comprising seven knowledge items, eight attitude items, and thirty-two skill items. The knowledge items are multiple-choice questions with a single correct answer, scoring 1 for a correct response and 0 for an incorrect response. The attitude and skill items employ a Likert-type five-point scale for scoring, ranging from 1 (strongly disagree/not confident at all) to 5 (strongly agree/very confident). To ensure the quality of the instrument, content validity was evaluated by six experts in the fields of family medicine, health education, and medical education. Experts assessed each item and simulated scenario for its adequacy, significance, and clarity, using a four-point scale. The Content Validity Index (CVI) was calculated by dividing the number of experts who rated an item as 3 or higher by the total number of experts. All items achieved a CVI of 0.80 or higher, indicating good content validity. Furthermore, internal consistency reliability was assessed using Cronbach’s α, resulting in a high reliability coefficient of 0.944, demonstrating excellent internal consistency of the scale. An English version of the questionnaire is provided as a supplementary file.

### Rationale and handling of pre-test in the experimental design

Although the study followed a true experimental design, a pre-test was included for two primary purposes: (1) to confirm the baseline equivalence between the experimental and control groups, and (2) to measure within-group improvement across the intervention. The use of a pre-test enabled the calculation of learning gains and further supported the analysis using Generalized Estimating Equations (GEE).

Importantly, both the experimental and control groups used the same validated questionnaire for the pre- and post-tests, and no feedback or correct answers were provided after the pre-test. Statistical analyses showed no significant pre-test differences between the two groups, minimizing concerns about pre-test sensitization or bias. This approach ensured the internal validity of the study while allowing for robust comparison of learning outcomes.

### Statistical analysis

Upon collecting and organizing the pre-test and post-test questionnaires, we conducted a statistical analysis using SPSS version 22 (IBM SPSS Statistics for Windows, Armonk, NY: IBM Corp). Descriptive statistics were employed to describe the essential information and distribution of participants. Pearson’s chi-square test was applied to examine potential associations between categorical variables (e.g., gender and school) and baseline health literacy scores, to ensure the comparability of groups prior to intervention [30]. A paired t-test was used to analyze the differences in the knowledge, attitude, and skill items within each group before and after the test. These items were measured using the average score per item. Moreover, we employed generalized estimating equations (GEE) to compare the intervention effects among different groups. GEE is particularly well-suited for analyzing repeated measurements, accommodating discrete and continuous correlated responses [31]. It provides an unbiased estimation of population-averaged regression coefficients, even in the presence of potential misspecification of the correlation structure. In our analysis, we considered the timing (pre- or post-test), group differences, and the interaction between time and group as sources of variation when measuring the disparity in knowledge, attitude, and skill items between two groups within the intervention.

## Results

The average age of 5th-year medical students was 23.3 years, with 72% males and 28% females. A total of 216 students completed both pre- and post-test questionnaires, comprising the experimental group (*n* = 110) and control group (*n* = 106). Pearson’s chi-square tests indicated no statistically significant differences for gender (knowledge: *p* = 0.145, attitude: *p* = 0.232, skill: *p* = 0.595) and school (knowledge: *p* = 0.171, attitude: *p* = 0.238, skill: *p* = 0.515) variables. Thus, the background characteristics of the two groups were homogeneous and comparable.

Generalized estimating equations (GEE) were employed to evaluate the effects of the intervention between the two groups, revealing an interaction between group and time. In the experimental group, the pre-test accuracy on cognitive variables was 0.8% higher than that of the control group. In contrast, attitude variables were 0.04 points lower, and skill variables were 0.046 points lower. However, none of these differences were statistically significant, confirming the homogeneity of the two groups at the pre-test stage.

Regarding the primary effect of “time,” the control group showed improvements in post-test scores compared to pre-test scores, with a 3% increase in cognitive variables, a 0.152 increase in attitude variables, and a 0.292 increase in skill variables (Table 2).

**Table 2:** GEE analyses with comparisons of pre-test and post-test between two groups

Item	𝛽	Std. Error	95% CI	OR	OR 95% CI	*P*
**Knowledge**						
Intercept	0.569	0.017	0.535 ~ 0.603			0.000
Group (ref.=control group)	0.008	0.024	-0.04 ~ 0.056			0.748
Time (ref.=pre-test)	0.03	0.016	-0.002 ~ 0.061			0.066
Time*Group	0.117	0.024	0.07 ~ 0.164	1.12	1.07 ~ 1.18	0.000
**Attitude**						
Intercept	4.539	0.045	4.451 ~ 4.627			0.000
Group (ref.=control group)	-0.04	0.060	-0.158 ~ 0.078			0.507
Time (ref.=pre-test)	0.152	0.04	0.074 ~ 0.231			0.000
Time*Group	0.175	0.057	0.063 ~ 0.287	1.19	1.07 ~ 1.33	0.002
**Skills**						
Intercept	4.256	0.055	4.149 ~ 4.364			0.000
Group (ref.=control group)	-0.046	0.075	-0.192 ~ 0.101			0.541
Time (ref.=pre-test)	0.292	0.048	0.199 ~ 0.386			0.000
Time*Group	0.162	0.073	0.019 ~ 0.305	1.18	1.02 ~ 1.36	0.026

When considering the combined effects of group and time on the instructional intervention, significant differences were observed in the improvement rates between the two groups. The experimental group showed an 11.7% increase in cognitive variables, a 0.175 increase in attitude variables, and a 0.162 increase in skill variables compared to the control group. The p-values for all three variables were less than 0.05 (Table 2), indicating interactions between the two groups and the two-time points.

To aid interpretation, we clarify that the β coefficients obtained from the GEE model represent the marginal effects—indicating the average change in the outcome variable (knowledge, attitude, or skill score) for a one-unit change in the predictor, assuming all other variables are held constant. Since all outcome variables in this study were continuous, these coefficients should be interpreted as mean differences, rather than traditional standardized effect sizes. For ease of interpretation, we additionally converted the β coefficients of the interaction term (Time*Group) into odds ratios (ORs): knowledge (OR = 1.12, 95% CI = 1.07–1.18), attitude (OR = 1.19, 95% CI = 1.07–1.33), and skills (OR = 1.18, 95% CI = 1.02–1.36). These ORs indicate that, on average, the intervention group was significantly more likely to demonstrate improvements across all three domains after the intervention.

**Fig. 2:**
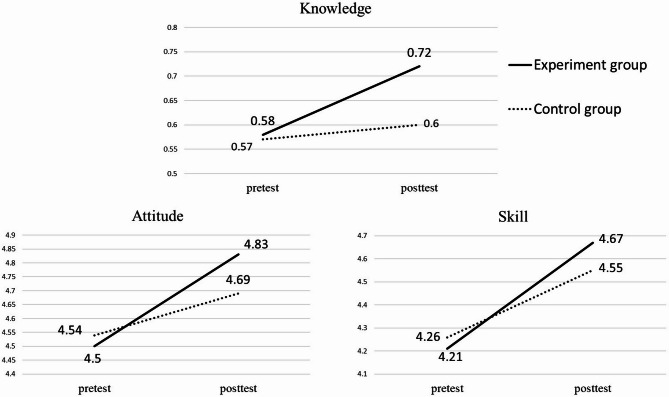
The difference in the average scores of three variables between two groups

Paired t-tests were conducted to separate the effects of the intervention on each variable within two groups, and the results are presented in Table 3. The experimental group showed significant improvements across all three variables, with post-test scores consistently higher than pre-test scores. While the control group also showed significant improvements in attitude and skills, the extent of improvement in all three variables was smaller than that observed in the experimental group (Fig. 2). The slopes of the knowledge, attitude, and skill variables were higher in the experimental group (1.02, 0.33, and 0.46, respectively) compared to the control group (0.21, 0.15, and 0.29, respectively). These findings indicate that the experimental group achieved significant advancements in all measured aspects. Therefore, it can be concluded that the “Innovative Teaching Module with Clinical Scenario-Integrated Interactive E-Book” intervention significantly enhanced the overall health literacy of medical students.

**Table 3:** The effect of intervention within each group

Variable	Experiment group (*n* = 110)			Control group (*n* = 106)		
	Pre-test Mean (SD)	Post-test Mean (SD)	*P*	Pre-test Mean (SD)	Post-test Mean (SD)	*P*
Knowledge (range:0–1)	0.58 (0.18)	0.72 (0.19)	0.000	0.57 (0.18)	0.60 (0.16)	0.070
Attitude (range:1–5)	4.50 (0.42)	4.83 (0.32)	0.000	4.54 (0.47)	4.69 (0.46)	0.000
Skill (range:1–5)	4.21 (0.53)	4.67 (0.44)	0.000	4.26 (0.57)	4.55 (0.53)	0.000

## Discussion

Digital learning will be one of the most important educational methods in the future. E-learning in medical education has gained popularity and rapidly developed following the onset of the COVID-19 pandemic [32–36]. A systematic review conducted in accordance with PRISMA guidelines examined 42 studies and revealed that the foremost e-learning modalities were interactive, asynchronous, and accessible from home [37]. The benefits encompass convenience [34–36] and diverse e-content featuring multimedia, animations, simulations, interactive activities [38], voiceovers, quizzes, and games. However, drawbacks include issues related to internet stability and security [35, 36, 38], eye strain [34], lack of concentration [34, 36], and comparatively lower academic performance when compared to traditional on-site learning [32]. Building upon aforementioned advantages, this study integrates a vast array of simulated clinical scenario videos, teacher (Visiting Staff, VS) narrated explanations, and engaging game-based learning techniques to develop an electronic book to enhance student self-learning motivation in medical education. This initiative enables aspiring clinicians to transcend temporal and spatial constraints. The “Interactive E-book on Clinical Scenarios in Health Literacy” is accessible anytime, anywhere, through mobile applications or the Hamastar website.

This is the first study in Taiwan to evaluate the effects of an interactive e-book as an e-learning material on simulative clinical scenarios in health literacy. The e-book’s efficacy was observed to significantly improve students’ understanding, attitudes, and skills related to health literacy. This is similar to the results of two studies collaborating with Hamastar Technology to develop e-books. One of them is the researchers conducted an educational intervention on blood and bone marrow cell morphology with 41 medical students [39]. They let twenty-five students utilize slides, while the remaining 26 utilized an interactive e-book. After the intervention, the students who read the e-book exhibited significant acceptance of the e-book, strong positive interest, motivation, and improved learning outcomes [39]. The other study involved the researchers randomly assigned 66 nursing students into two groups on average [40]. In the sexual harassment course, the experiment group used an e-book, and the control group applied video and brochure. The knowledge, motivation, and prevention strategies of the experiment group were significantly higher than the control group [40]. These studies, such as ours, have demonstrated the beneficial impact of interactive e-books on learning outcomes.

Physicians with adequate health literacy skills can significantly influence their patients’ well-being. Understanding how to assess patients’ health literacy levels enables selecting the most suitable communication approach, facilitating patient comprehension of treatment and associated medical information. This, in turn, fosters collaborative discussions between patients and physicians to arrive at optimal medical decisions tailored to individual needs, ultimately enhancing well-being. Accessible digital educational resources provide valuable self-learning opportunities for busy medical students and practitioners. This study introduces an engaging e-book featuring interactive clinical scenarios designed to enhance health literacy. This resource holds promise as a teaching adjunct in health literacy courses within medical education, inspiring improved patient-physician interactions and better health outcomes for patients. Furthermore, it is a self-learning tool to foster communication between patients and physicians, offering a hopeful vision of enhanced healthcare delivery.

### Limitation

This study is a cross-sectional research with two measurement timings, limiting the exploration of sustainability of intervention effectiveness. Introducing retention tests three or six months following the intervention could introduce confounding biases. Additionally, although subgroup analyses based on gender and school were performed and showed no significant baseline differences, this study did not assess prior experience with digital learning tools, limiting further exploration of this potential moderator. Extensive subgroup analyses were also avoided to prevent multiple-testing issues. Moreover, the control group did not receive additional learning activities of equivalent duration, so the difference in time-on-task between groups should be considered when interpreting the observed improvements. Hence, future research endeavors may consider conducting experiments involving students with similar characteristics from different schools, with one school designated as the experimental group and another as the control group, to mitigate potential interference and further investigate demographic or experiential factors affecting outcomes.

## Conclusion

This study underscores the transformative potential of interactive e-books featuring simulative clinical scenarios in advancing health literacy competencies among medical students. By integrating multi-theories and game-based learning strategies, the e-book significantly enhanced students’ knowledge, attitudes, and skills critical for effective patient-centered care. The results demonstrate that such innovative digital learning tools promote student engagement and prepare future physicians to navigate complex patient interactions with empathy, clarity, and precision. The marked improvement observed in the experimental group highlights the efficacy of incorporating technology-driven educational resources into medical curricula. These findings emphasize the importance of equipping medical students with the requisite competencies to address diverse patient needs, foster collaborative decision-making, and enhance healthcare outcomes. Future research should examine the long-term impact and scalability of these interventions across various medical specialties to further enrich the field of medical education and healthcare delivery.

## Electronic supplementary material

Below is the link to the electronic supplementary material.

Supplementary Material 1

## Data Availability

The datasets generated and analyzed during the current study are available from the corresponding author on reasonable request.

## Declarations

### Author constributions

P-L T. led the conceptualization of the study alongside C-C C., J-S T., H-R L., and C-Y T. Additionally, P-L T., H-F Y., and C-Y T. collaborated on curriculum development, ensuring the course met the necessary standards. Data curation was managed by P-L T., H-F Y., and C-C C., who organized and prepared the data for analysis. P-L T. and H-F Y. conducted the formal analysis, ensuring the integrity of the findings. The administration of the project was overseen by P-L T. and T-Y C., ensuring smooth project execution. Finally, the original draft of the manuscript was primarily written by P-L T. and C-Y T., who synthesized the research into a cohesive document. All authors reviewed the manuscript.

## References

[1] Duong H, Chang P. Topics included in health literacy studies in asia: A systematic review. Asia Pac J Public Health. 2024;36(1):8–19. 38156482 10.1177/10105395231220473

[2] Baccolini V, Isonne C, Salerno C, Giffi M, Migliara G, Mazzalai E et al. The association between adherence to cancer screening programs and health literacy: A systematic review and meta-analysis. 2022;155:106927. 10.1016/j.ypmed.2021.106927 34954244

[3] Lin C-W, Ho C-J, Huang R-Y, Wang W-D. Health literacy: conceptual development and practical application. Taiwan J Family Med. 2016;26(2):65–76.

[4] Coleman CA, Appy S. Health literacy teaching in US medical schools, 2010. Fam Med. 2012;44(7):504–7. 22791536

[5] Coleman CA, Fromer A. A health literacy training intervention for physicians and other health professionals. Fam Med. 2015;47(5):388–92. 25905883

[6] Coleman CA, Peterson-Perry S, Bumsted T. Long-term effects of a health literacy curriculum for medical students. Fam Med. 2016;48(1):49–53. 26950666

[7] Hung C-T, Wu S-E, Chen Y-H, Soong C-Y, Chiang CP, Wang WM. The evaluation of synchronous and asynchronous online learning: student experience, learning outcomes, and cognitive load. BMC Med Educ. 2024;24(1):326. 38519950 10.1186/s12909-024-05311-7 PMC10960437

[8] Yamada R, Futakawa K, Xu K, Kondo S. Using virtual patients to enhance empathy in medical students: a scoping review protocol. Syst Reviews. 2025;14(1):52. 10.1186/s13643-025-02793-4 PMC11871709 40025554

[9] Bhavsar MH, Javia HN, Mehta SJ. Flipped classroom versus traditional didactic classroom in medical teaching: a comparative study. Cureus. 2022;14(3). 10.7759/cureus.23657 PMC9060739 35510025

[10] Ji M, Luo Z, Feng D, Xiang Y, Xu J. Short-and long-term influences of flipped classroom teaching in physiology course on medical students’ learning effectiveness. Front Public Health. 2022;10:835810. 35419334 10.3389/fpubh.2022.835810 PMC8995769

[11] Chandran VP, Balakrishnan A, Rashid M, Pai Kulyadi G, Khan S, Devi ES, et al. Mobile applications in medical education: A systematic review and meta-analysis. PLoS ONE. 2022;17(3):e0265927. 35324994 10.1371/journal.pone.0265927 PMC8947018

[12] Wynter L, Burgess A, Kalman E, Heron JE, Bleasel J. Medical students: what educational resources are they using? BMC Med Educ. 2019;19(1):36. 30683084 10.1186/s12909-019-1462-9 PMC6347772

[13] Turner KL, Chung H. Transition to eBook provision: A commentary on the preferences and adoption of eBooks by chemistry undergraduates. J Chem Educ. 2020;97(5):1221–5.

[14] White S, Chen J, Forsyth B. Reading-related literacy activities of American adults: time spent, task types, and cognitive skills used. J Lit Res. 2010;42(3):276–307.

[15] Chang C-C, Lin K-Y. From traditional e-Learning to digital Game-Based learning: learning performance, flow experience and cognitive load. Contemp J Sci Educ. 2016;24(3):221–48.

[16] Li C-J, Su H-C, Chen-Pao-Ju, Pan W-L, Hsiao C-L, Hu SH, et al. The learning effectiveness of innovative Game-Based learning on high alert medications use among new nurses. New Taipei Jouranl Nurs. 2019;21(2):1–11.

[17] Huang H-L, Chen M-P, Chiu P-H. The effects of Inquiry-Based gaming strategies and Self-Regulation on Sixth-Graders’ performance in learning influenza prevention concepts. Int J Diagital Learn Technol. 2012;4(3):1–15.

[18] Røe Y, Torbjørnsen A, Stanghelle B, Helseth S, Riiser K. Health literacy in higher education: a systematic scoping review of educational approaches. Pedagogy Health Promotion. 2025;11(1):20–9.

[19] Chuang S-T, Liao P-L, Lo S-F, Chang Y-T, Hsu H-T. Effectiveness of an E-book app on the knowledge, attitudes and confidence of nurses to prevent and care for pressure injury. Int J Environ Res Public Health. 2022;19(23):15826. 36497905 10.3390/ijerph192315826 PMC9737897

[20] Yu T-Y, Huang T-W, Huang H-C, Li S-Y, Chuang Y-H. Effects of an interactive e-Book on enhancing nursing students’ knowledge, confidence, and learning Self-efficacy of nursing skills: A randomized controlled trial. Nurse Educ. 2024;49(1):E20–5. 37647544 10.1097/NNE.0000000000001490

[21] Hsiao Y-T, Liu H-Y, Hsiao C-C, editors. Development of a novel interactive multimedia e-learning model to enhance clinical competency training and quality of care among medical students. Healthcare: MDPI; 2020. 10.3390/healthcare8040500 PMC7712745 33233509

[22] Phillips BC, Johnson J, Khalid N, Zapparrata N, Albright G. Benefits of an online interactive educational program over traditional textbooks. Nurse Educ. 2023;48(5):270–5. 36881473 10.1097/NNE.0000000000001398 PMC10467810

[23] Dong ZY, Zhang Y, Yip C, Swift S, Beswick K. Smart campus: definition, framework, technologies, and services. IET Smart Cities. 2020;2(1):43–54.

[24] Lin C-Y, Lin Y-J, Mao J-J, Lin F-T, Chen C-T, Liu H-R, et al. Implementation overview of Non-Pharmaceutical interventions for COVID-19 in Taiwan. Taiwan Epidemiol Bull. 2024;40(18):276–90.

[25] Yang H-F, Chang C-C, Tseng P-L, Lai H-R, Tasi J-S, Huang W-H, et al. Effectiveness of innovative instructional module for professional competence in health literacy in medical students. BMC Med Educ. 2022;22(1):210. 35351115 10.1186/s12909-022-03252-7 PMC8960696

[26] Mayer RE, Moreno R. Nine ways to reduce cognitive load in multimedia learning. Educational Psychol. 2003;38(1):43–52.

[27] Trochim WMJE. planning p. An introduction to concept mapping for planning and evaluation. 1989;12(1):1–16.

[28] Reigeluth CM. Instructional design theories and models: an overview of their current status. Routledge; 1983.

[29] Liu CC, Chang CC, Lai HR, Tsai JS, Ming JL, Tung CY. Study of constructing indicators of physician’s professional competence on health literacy by Delphi method. Taiwan J Public Health. 2020;39(3):292–310.

[30] Schober P, Vetter TR. Chi-square tests in medical research. Anesth Analgesia. 2019;129(5):1193. 10.1213/ANE.0000000000004410 31613806

[31] Wang M. Generalized estimating equations in longitudinal data analysis: a review and recent developments. Adv Stat. 2014;2014(1):303728.

[32] Varachotisate P, Siritaweechai N, Kositanurit W, Thanprasertsuk S, Chayanupatkul M, Thongsricome T, et al. Student academic performance in non-lecture physiology topics following the abrupt change from traditional on-site teaching to online teaching during COVID-19 pandemic. Med Educ Online. 2023;28(1):2149292. 36419226 10.1080/10872981.2022.2149292 PMC9704068

[33] Rastogi A, Bansal A, Keshan P, Jindal A, Prakash A, Kumar V. Medical education in post-pandemic times: online or offline mode of learning? J Family Med Prim Care. 2022;11(9):5375–86. 36505568 10.4103/jfmpc.jfmpc_2305_21 PMC9731027

[34] Singh A, Jadon RS, Baitha U, Sethi P, Kaur H, Kumar A, et al. Indian medical student perspectives on online mode of education. J Family Med Prim Care. 2022;11(7):3915–22. 36387726 10.4103/jfmpc.jfmpc_2158_21 PMC9648222

[35] Syed S, Rastogi A, Bansal A, Kumar A, Jindal A, Prakash A, et al. editors. Future of e-learning in medical education—perception, readiness, and challenges in a developing country. Frontiers in Education; 2021: Frontiers Media SA.

[36] Padhi KS, Balmuchu G, Acharya PS, Singh SR, Joseph T. The perspectives of educators and learners on E-learning: a cross-sectional descriptive study in a medical school. Adv Med Educ Pract. 2021:1059–66. 10.2147/AMEP.S326147 PMC8464331 34584482

[37] Delungahawatta T, Dunne S, Hyde S, Halpenny L, McGrath D, O’Regan A, et al. Advances in e-learning in undergraduate clinical medicine: a systematic review. BMC Med Educ. 2022;22(1):711. 36207721 10.1186/s12909-022-03773-1 PMC9540295

[38] Bankar MN, Bankar NJ, Singh BR, Bandre GR, Shelke YP, Bankar M et al. The role of E-Content development in medical teaching: how Far have we come?? Cureus. 2023;15(8). 10.7759/cureus.43208 PMC10488137 37692742

[39] Hsiao C-C, Tiao M-M, Chen C-C. Using interactive multimedia e-Books for learning blood cell morphology in pediatric hematology. BMC Med Educ. 2016;16:1–8. 27842530 10.1186/s12909-016-0816-9 PMC5109786

[40] Chang T-S, Teng Y-K, Chien S-Y, Tzeng Y-L. Use of an interactive multimedia e-book to improve nursing students’’sexual harassment prevention knowledge, prevention strategies, coping behavior, and learning motivation: A randomized controlled study. Nurse Educ Today. 2021;105:104883. 34218069 10.1016/j.nedt.2021.104883

