# Supplementary material for: The effect of an interactive game-based e-book with simulative clinical scenarios on the health literacy competency among medical students in Taiwan
Source: BMC Med Educ. 2025 Jul 1;25:876. doi: 10.1186/s12909-025-07541-9 (PMC12211249; doi:10.1186/s12909-025-07541-9)
Supplement: Supplementary file 1 — Supplementary Material 1 [file 12909_2025_7541_MOESM1_ESM.docx]

**Physician Health Literacy and Professional Competence Questionnaire**

Dear Physicians/Prospective Physicians,

This is a research project survey aimed at understanding your perspectives on patient health literacy, the provision of medical information and physician-patient communication.

For statistical analysis purposes, data will be collected at two different points in times. Your responses will only be used solely for academic research and will remain strictly confidential. Kindly answer based on your actual thoughts and ensure that no questions are left unanswered.

Thank you for your assistance!

Best wishes,

Dr. Cheng-Yin Tung, Professor

MS. and MPH. Pei-Ling Tseng, Research Assistant

Department of Health Promotion and Health Education,

National Taiwan Normal University

Contact Number: 886-2-7749-1730

March, 2021

**【Section 1: Basic Information】**

1. Gender: □ Male □ Female
2. Age:
3. Grade: □ First □ Second □ Third □ Fourth □ Fifth □ Sixth
4. Your School: □ National Taiwan University □ Chang Gung University
   □Taipei Medical University □ National Yang Ming Chiao Tung University
   □ Kaohsiung Medical University □ Chung Shan Medical University
   □ China Medical University □ Tzu Chi University □ National Cheng Kung University □ Mackay Medical College □ Fu Jen Catholic University
   □ National Defense Medical Center
5. Last 5 digits of your student ID (for duplicate response verification):

**【Section 2: Conceptual Understanding】**

1. Which of the following is the most complete definition of health literacy?

□ The ability to obtain, understand and apply medical information

□ The ability to follow the medical instructions and take drugs on time

□ The ability to recognize one’s own physical discomfort

□ The ability to choose the appropriate medical department for consultation

2. Which of the following is NOT a tool used to assess patient health literacy?

□ Mandarin Multidimensional Health Literacy Questionnaire

□ Brief Symptom Rating Scale (BSRS-5)

□ Newest vital sign scores

□ Taiwan Health Literacy Scale (THLS)

【Section 3: Physician-Patient Interaction Scenarios】

In this section, you will be asked to choose the answer that best fits your thoughts.

Scenario 1

Mr. Wang, an 82-year-old Minnan-speaking senior with an incomplete elementary education, listens to radio programs daily and regularly purchases health supplements advertised on the radio. Recently, he felt discomfort while urinating and suspected a kidney problem, prompting him to seek medical attention. Upon entering a crowded clinic, he chose to sit in a corner and only entered the consultation room when his number was displayed. When the doctor asked, “How do you feel today?” Mr. Wang became nervous, lowered his head, and remained silent for a long time.

1. Which of the following is NOT a sign of Mr. Wang’s inadequate health literacy?

□ Being 82 years old with an incomplete elementary education

□ Regularly listening to radio programs and purchasing supplements

□ Choosing to visit a nephrologist due to urinary discomfort

□ Feeling nervous and silent in front of the doctor

1. It is important for doctors to recognize and assess Mr. Wang's level of health literacy.

□ Strongly agree □ Agree □ Neutral □ Disagree □ Strongly disagree

1. If doctors can provide services based on Mr. Wang's health literacy, it will help improve the quality of medical services and reduce the waste of healthcare resources.

□ Strongly agree □ Agree □ Neutral □ Disagree □ Strongly disagree

1. Based on my clinical experience and the current healthcare environment, if I were Mr. Wang's physician, I would adopt an appropriate physician-patient interaction model according to his health literacy.

□ Very confident □ Confident □ Somewhat confident □ Not confident □ Not confident at all

1. Based on my clinical experience and the current healthcare environment, if I were Mr. Wang's physician, I would be able to arrange a comfortable communication environment for him.

□ Very confident □ Confident □ Somewhat confident □ Not confident □ Not confident at all

1. Based on my clinical experience and the current healthcare environment, if I were Mr. Wang's physician, I would be able to create a relaxed atmosphere, making Mr. Wang feel that the doctor is approachable.

□ Very confident □ Confident □ Somewhat confident □ Not confident □ Not confident at all

1. Based on my clinical experience and the current healthcare environment, if I were Mr. Wang's physician, I would first confirm his medical history, lifestyle habits, and financial situation during the consultation.

□ Very confident □ Confident □ Somewhat confident □ Not confident □ Not confident at all

1. Based on my clinical experience and the current healthcare environment, if I were Mr. Wang's physician, I would be able to use or seek assistance to communicate with him in Minnan, the language he is familiar with, to explain his medical condition.

□ Very confident □ Confident □ Somewhat confident □ Not confident □ Not confident at all

1. Based on my clinical experience and the current healthcare environment, if I were Mr. Wang's physician, I would appropriately use gestures or eye contact to help him better understand his medical condition.

□ Very confident □ Confident □ Somewhat confident □ Not confident □ Not confident at all

1. Based on my clinical experience and the current healthcare environment, if I were Mr. Wang's physician, I would use questioning techniques to guide him in expressing his actual medical concerns and condition.

□ Very confident □ Confident □ Somewhat confident □ Not confident □ Not confident at all

Scenario 2

Mrs. Lin, 75 years old, has recently experienced chest discomfort and is worried after hearing media reports about seniors dying of heart attacks in cold weather. She rushed into the consultation room before the previous patient had left and exclaimed, “Doctor, I heard that the elderly are prone to heart attacks. What is a heart attack? It sounds terrifying! My neighbor recently died suddenly from heart problems. I also saw on TV that pricking the finger to release blood can help. Recently, I’ve felt chest tightness when lifting objects. Please check my body immediately. Do I get a heart attack?”

1. Which of the following is NOT a sign of Mrs. Lin’s inadequate health literacy?

□ Hearing reports that the incidences of heart attack increase due to cold weather

□ Not following the proper appointment sequence

□ Believing media reports that pricking fingers can prevent heart attacks

□ Excessively worrying about having a heart attack

1. Which factor affects Mrs. Lin's ability to make correct and appropriate decisions and actions in her daily life and medical visits?

□ Health literacy

□ Life skills

□ General knowledge

□ Civic literacy

1. If the doctor can provide services based on Mrs. Lin's ability to obtain, comprehend, and apply medical information, it will be helpful in establishing a good physician-patient relationship.

□ Strongly agree □ Agree □ Neutral □ Disagree □ Strongly disagree

1. Doctor should empathize with and accept Mrs. Lin's anxiety about myocardial infarction.

□ Strongly agree □ Agree □ Neutral □ Disagree □ Strongly disagree

1. Doctor should invite Mrs. Lin's family to join her in the consultation room to provide her with more support and confidence.

□ Strongly agree □ Agree □ Neutral □ Disagree □ Strongly disagree

1. Doctor should patiently listen to Mrs. Lin's statements without interrupting her.

□ Strongly agree □ Agree □ Neutral □ Disagree □ Strongly disagree

1. Based on my clinical experience and the current healthcare environment, if I were Mrs. Lin's physician, I would ask Mrs. Lin, who suddenly rushed into the consultation room, to wait outside in order to protect the privacy of the previous patient.

□ Very confident □ Confident □ Somewhat confident □ Not confident □ Not confident at all

1. Based on my clinical experience and the current healthcare environment, if I were Mrs. Lin's physician, I would use simple words to replace medical terms such as myocardial infarction and atherosclerosis when explaining the condition to her.

□ Very confident □ Confident □ Somewhat confident □ Not confident □ Not confident at all

1. Based on my clinical experience and the current healthcare environment, if I were Mrs. Lin's physician, I would provide her with no more than three new medical concepts at a time.

□ Very confident □ Confident □ Somewhat confident □ Not confident □ Not confident at all

1. Based on my clinical experience and the current healthcare environment, if I were Mrs. Lin's physician, I would proactively clarify her misconception about treating a heart attack by pricking the finger to release blood.

□ Very confident □ Confident □ Somewhat confident □ Not confident □ Not confident at all

1. Based on my clinical experience and the current healthcare environment, if I were Mrs. Lin's physician, I would be able to provide appropriate educational materials (paper or video) to help her accurately understand myocardial infarction and its causes.

□ Very confident □ Confident □ Somewhat confident □ Not confident □ Not confident at all

1. Based on my clinical experience and the current healthcare environment, if I were Mrs. Lin's physician, I would be able to patiently discuss with her the necessary medical examinations based on her preferences and concerns.

□ Very confident □ Confident □ Somewhat confident □ Not confident □ Not confident at all

Scenario 3

Ms. Ruan, a Vietnamese immigrant who has lived in Taiwan for over a decade, recently attended a water activity with her child. The next day, she developed red and swollen eyes with persistent discharge. She visited an ophthalmologist with her husband, who helped fill out the registration form because she has limited knowledge of Chinese characters. The doctor diagnosed her with acute viral conjunctivitis and recommended using eye drops while also advising her to wash her hands with soap after each application. Confused, she asked, “Isn’t using the eye drops enough? Why do I need to wash my hands with soap?”

1. Which of the following is NOT a sign of Mrs. Ruan’s inadequate health literacy?

□ Being a Vietnamese immigrant

□ Having her husband accompany her to the ophthalmologist

□ Being unable to fill out the registration form by herself

□ Seeking medical attention for red and swollen eyes

1. Doctor should adopt an inclusive attitude in accepting Ms. Ruan, who comes from a different cultural background.

□ Strongly agree □ Agree □ Neutral □ Disagree □ Strongly disagree

1. Based on my clinical experience and the current healthcare environment, if I were Mrs. Ruan's physician, I would be able to assist her in understanding conjunctivitis and help her correctly apply eye drops and wash her hands with soap.

□ Very confident □ Confident □ Somewhat confident □ Not confident □ Not confident at all

1. Based on my clinical experience and the current healthcare environment, if I were Mrs. Ruan's physician, I would be able to allocate sufficient time for guidance and communication with her, sincerely providing information to gain her trust.

□ Very confident □ Confident □ Somewhat confident □ Not confident □ Not confident at all

1. Based on my clinical experience and the current healthcare environment, if I were Mrs. Ruan's physician, I would be able to slow down my speech and use an appropriate volume when communicating with her.

□ Very confident □ Confident □ Somewhat confident □ Not confident □ Not confident at all

1. Based on my clinical experience and the current healthcare environment, if I were Mrs. Ruan's physician, I would be able to discuss the treatment goals with her based on her condition of acute viral conjunctivitis.

□ Very confident □ Confident □ Somewhat confident □ Not confident □ Not confident at all

1. Based on my clinical experience and the current healthcare environment, if I were Mrs. Ruan's physician, after explaining the correct method of using the eye drops, I would choose an appropriate time to have her repeat the instructions in her own words to ensure she fully understands.

□ Very confident □ Confident □ Somewhat confident □ Not confident □ Not confident at all

1. Based on my clinical experience and the current healthcare environment, if I were Mrs. Ruan's physician, I would guide her in practicing the application of eye drops and provide feedback to ensure the procedure is performed correctly.

□ Very confident □ Confident □ Somewhat confident □ Not confident □ Not confident at all

1. Based on my clinical experience and the current healthcare environment, if I were Mrs. Ruan's physician, I be able to assess the accuracy of her eye drop application techniques and steps, and clarify or provide additional guidance on any mistakes.

□ Very confident □ Confident □ Somewhat confident □ Not confident □ Not confident at all

1. Based on my clinical experience and the current healthcare environment, if I were Mrs. Ruan's physician, I be able to appropriately express recognition or praise when she correctly applies the eye drops.

□ Very confident □ Confident □ Somewhat confident □ Not confident □ Not confident at all

1. Based on my clinical experience and the current healthcare environment, if I were Mrs. Ruan's physician, I make good use of her husband's support to enhance her willingness to follow medical instructions.

□ Very confident □ Confident □ Somewhat confident □ Not confident □ Not confident at all

Scenario 4

Mr. Liang, who has a history of chronic illnesses and long-term medication use, recently experienced numbness in his jaw and neck along with chest tightness. He was referred from the endocrinology department to cardiology. The cardiologist suspected coronary artery blockage and recommended cardiac catheterization. The doctor also informed him that if the blockage exceeded 70%, a stent might be required and asked him to choose a stent before the examination.

1. What is the possible outcome if Mr. Liang receives appropriate medical services based on his health literacy?

□ A decreased awareness of Mr. Liang’s health and motivation to seek treatment

□ Mr. Liang is unable to have accurate expectations regarding the prognosis of his illness.

□ Mr. Liang may have unrealistic medical expectations.

□ Mr. Liang can effectively utilize healthcare resources.

1. If the doctor can recognize and assess Mr. Liang's health literacy, and subsequently provide him with appropriate information on the prevention and treatment of coronary artery blockage, it would be highly valuable for the effective utilization of healthcare resources.

□ Strongly agree □ Agree □ Neutral □ Disagree □ Strongly disagree

1. Based on my clinical experience and the current healthcare environment, if I were Mrs. Liang 's physician, I would first discuss with him the department for referral, the doctor's expertise, and the consultation details, and obtain his consent before proceeding with the referral.

□ Very confident □ Confident □ Somewhat confident □ Not confident □ Not confident at all

1. Based on my clinical experience and the current healthcare environment, if I were Mrs. Liang 's physician, I would be able to clearly and specifically explain the treatment objectives and the possible future progression of his condition based on his clinical symptoms

□ Very confident □ Confident □ Somewhat confident □ Not confident □ Not confident at all

1. Based on my clinical experience and the current healthcare environment, if I were Mrs. Liang 's physician, I would use appropriate analogies, such as "a pipe that gets clogged over time," to explain coronary artery blockage.

□ Very confident □ Confident □ Somewhat confident □ Not confident □ Not confident at all

1. Based on my clinical experience and the current healthcare environment, if I were Mrs. Liang 's physician, I would be able to clearly explain the purpose of stent placement, the possible consequences of not having a stent, and encourage him to participate in the discussion.

□ Very confident □ Confident □ Somewhat confident □ Not confident □ Not confident at all

1. Based on my clinical experience and the current healthcare environment, if I were Mrs. Liang 's physician, I would allow him sufficient time to consider or discuss with his family whether to have a stent placed and to choose the type of stent, without pressuring him to make an immediate decision.

□ Very confident □ Confident □ Somewhat confident □ Not confident □ Not confident at all

1. Based on my clinical experience and the current healthcare environment, if I were Mrs. Liang 's physician, I would be able to provide him with an easy-to-read brochure or handbook about the cardiac catheterization procedure for him to use at home.

□ Very confident □ Confident □ Somewhat confident □ Not confident □ Not confident at all

1. Based on my clinical experience and the current healthcare environment, if I were Mrs. Liang 's physician, I would introduce heart disease patient group support to help him complete his treatment.

□ Very confident □ Confident □ Somewhat confident □ Not confident □ Not confident at all

1. Based on my clinical experience and the current healthcare environment, if I were Mrs. Liang 's physician, I would collaborate with other healthcare professionals to explain relevant disease information to him.

□ Very confident □ Confident □ Somewhat confident □ Not confident □ Not confident at all

1. Based on my clinical experience and the current healthcare environment, if I were Mrs. Liang 's physician, I would analyze the advantages and disadvantages of various stent options, including costs, risks, prognosis, and recovery timeline, enabling him to make an informed decision and be willing to take responsibility for his choice.

□ Very confident □ Confident □ Somewhat confident □ Not confident □ Not confident at all

1. Based on my clinical experience and the current healthcare environment, if I were Mrs. Liang 's physician, I would allow him to fully express his preferences regarding treatment options, respect his decision, and ensure that he signs the consent form before proceeding with the treatment.

□ Very confident □ Confident □ Somewhat confident □ Not confident □ Not confident at all
